# The global economic burden of COVID-19 disease: a comprehensive systematic review and meta-analysis

**DOI:** 10.1186/s13643-024-02476-6

**Published:** 2024-02-16

**Authors:** Ahmad Faramarzi, Soheila Norouzi, Hossein Dehdarirad, Siamak Aghlmand, Hasan Yusefzadeh, Javad Javan-Noughabi

**Affiliations:** 1 Department of Health Economics and Management, School of Public Health, Urmia University of Medical Sciences, Urmia, Iran; 2 Department of Medical Library and Information Science, School of Allied Medical Sciences, Tehran University of Medical Sciences, Tehran, Iran; 3 Social Determinants of Health Research Center, Mashhad University of Medical Sciences, Mashhad, Iran

**Keywords:** COVID-19, Cost of illness, Economics, Systematic review, Pandemics

## Abstract

**Background:**

The COVID-19 pandemic has caused a considerable threat to the economics of patients, health systems, and society.

**Objectives:**

This meta-analysis aims to quantitatively assess the global economic burden of COVID-19.

**Methods:**

A comprehensive search was performed in the PubMed, Scopus, and Web of Science databases to identify studies examining the economic impact of COVID-19. The selected studies were classified into two categories based on the cost-of-illness (COI) study approach: top-down and bottom-up studies. The results of top-down COI studies were presented by calculating the average costs as a percentage of gross domestic product (GDP) and health expenditures. Conversely, the findings of bottom-up studies were analyzed through meta-analysis using the standardized mean difference.

**Results:**

The implemented search strategy yielded 3271 records, of which 27 studies met the inclusion criteria, consisting of 7 top-down and 20 bottom-up studies. The included studies were conducted in various countries, including the USA (5), China (5), Spain (2), Brazil (2), South Korea (2), India (2), and one study each in Italy, South Africa, the Philippines, Greece, Iran, Kenya, Nigeria, and the Kingdom of Saudi Arabia. The results of the top-down studies indicated that indirect costs represent 10.53% of GDP, while the total estimated cost accounts for 85.91% of healthcare expenditures and 9.13% of GDP. In contrast, the bottom-up studies revealed that the average direct medical costs ranged from US $1264 to US $79,315. The meta-analysis demonstrated that the medical costs for COVID-19 patients in the intensive care unit (ICU) were approximately twice as high as those for patients in general wards, with a range from 0.05 to 3.48 times higher.

**Conclusions:**

Our study indicates that the COVID-19 pandemic has imposed a significant economic burden worldwide, with varying degrees of impact across countries. The findings of our study, along with those of other research, underscore the vital role of economic consequences in the post-COVID-19 era for communities and families. Therefore, policymakers and health administrators should prioritize economic programs and accord them heightened attention.

**Supplementary Information:**

The online version contains supplementary material available at 10.1186/s13643-024-02476-6.

## Background

Coronavirus disease 2019 (COVID-19) is a respiratory infection instigated by the severe acute respiratory syndrome coronavirus 2 (SARS-COV-2), first identified in Wuhan, China, in December 2019. The disease has since proliferated globally at an alarming rate, prompting the World Health Organization (WHO) to declare a pandemic on March 11, 2020 [1]. As of February 21, 2023, the global total of confirmed COVID-19 cases stands at 757,264,511, with a death toll of 6,850,594 [2].

Patients afflicted with COVID-19 exhibit a range of symptoms, including flu-like manifestations, acute respiratory failure, thromboembolic diseases, and organ dysfunction or failure [3]. Moreover, these patients have had to adapt to significant changes in their environment, such as relocating for quarantine purposes, remote work or job loss, and air-conditioning [4, 5].

The COVID-19 pandemic has imposed substantial direct and indirect costs on patients, families, healthcare systems, and communities. These costs fluctuate significantly based on socioeconomic factors, age, disease severity, and comorbidities [6, 7]. For instance, a study conducted in the United States of America (USA) estimated the median direct medical cost of a single symptomatic COVID-19 case to be US $3045 during the infection period alone [8]. Additionally, indirect costs arising from the pandemic, such as lost productivity due to morbidity and mortality, reduced consumer spending, and supply chain disruptions, could be substantial in certain countries [9]. Studies by Maltezou et al. and Faramarzi et al. revealed that absenteeism costs accounted for a large proportion (80.4%) of total costs [10] and estimated an average cost of US $671.4 per patient [11], respectively. Furthermore, the macroeconomic impact of the COVID-19 pandemic is considerably more significant. Data from Europe indicates that the gross domestic product (GDP) fell by an average of 7.4% in 2020 [12]. Globally, the economic burden of COVID-19 was estimated to be between US $77 billion and US $2.7 trillion in 2019 [13]. Another study calculated the quarantine costs of COVID-19 to exceed 9% of the global GDP [14].

Evaluating the cost of COVID-19, encompassing both direct (medical and non-medical) and indirect costs, provides valuable insights for policymakers and healthcare managers to devise effective strategies for resource allocation and cost control, particularly in the post-COVID-19 era. Despite the abundance of literature on COVID-19, only a handful of studies have concentrated on its economic burden. Furthermore, the currency estimates provided in these articles is inconsistent. To address this gap, our study aimed to conduct a systematic review and meta-analysis of the global economic burden of COVID-19. The objectives of this study are twofold: firstly, to estimate the direct and indirect costs of COVID-19 as a percentage of GDP and health expenditure (HE) at the global level, and secondly, to estimate the direct medical costs based on the inpatient ward, which includes both the general ward and the intensive care unit (ICU).

## Methods

This study was designed according to the Preferred Reporting Items for Systematic Reviews and Meta Analyses (PRISMA) guidelines [15].

### Search strategy and data sources

We performed a comprehensive search in PubMed, Scopus, and Web of Science databases to retrieve studies on the economic burden of COVID-19 disease. To this objective, we conducted a comprehensive search by combining the search terms relating to COVID-19 (coronavirus, 2019-nCoV), as a class, with the terms relating to the economic burden and terms related to it (direct cost, indirect cost, productivity cost, morbidity cost, mortality cost, cost analysis, cost of illness, economic cost, noneconomic cost, financial cost, expenditure, spending). The search was limited to English language publications and human studies that were published before September 19, 2021. The search strategy was validated by a medical information specialist. All search strategies are available in the Additional file 1.

### Screening and selection

**Fig. 1:**
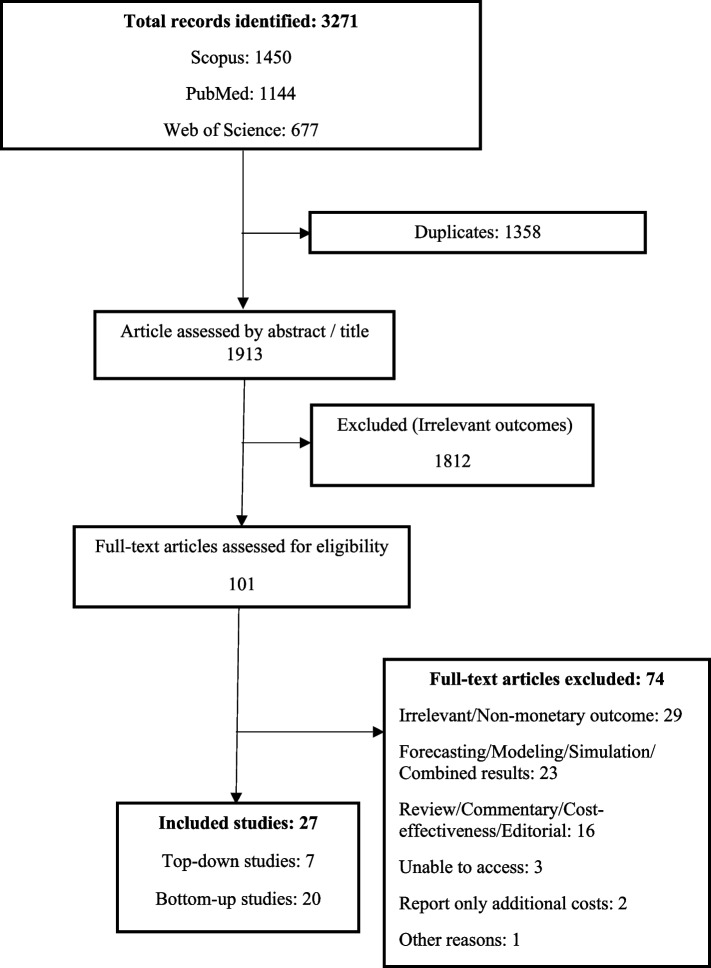
Flowchart depicting the selection of research studies

Two reviewers independently screened all distinct articles, focusing on the title and abstract and utilizing EndNote software. The reviewers were blinded to each other’s findings during the screening phase. Potential duplicates were identified and scrutinized to exclude identical entries. Any discrepancies between the reviewers were reconciled through consensus or by consulting a third reviewer. The final decision regarding inclusion was determined subsequent to a comprehensive review of the full-text article. The whole process of the study selection was outlined in a flow chart (Fig. 1).

This systematic review included all original studies that addressed the economic burden of COVID-19, provided they (1) estimated all costs associated with COVID-19, including both direct (medical and non-medical) and indirect (morbidity and mortality) costs and (2) were designed as observational studies or controlled clinical trials. Studies were excluded based on the following criteria: (1) they were review articles, commentaries, editorials, protocols, case studies, case series, animal studies, book chapters, or theses, (2) they estimated costs for a specific disease or action during the COVID-19 pandemic, and (3) they were studies assessing budget impact or economic evaluations.

### Data extraction

A specific data extraction template was developed to extract relevant information from every study that satisfied our eligibility criteria. The data extracted covered the general study characteristics (authors, study publication, geographical location of data collection), cost-related information (direct medical cost, direct nonmedical cost, indirect cost, total cost, years of costing, and currency), and participants-related data (sample size and population studied for estimation).

### Outcome and quality assessment

The primary outcomes were documented as the standardized mean difference (SMD) accompanied by 95% confidence intervals, representing the direct medical costs borne in general wards as compared to ICU for patients diagnosed with COVID-19. Additionally, another outcome was the estimation of these costs as a proportion of the GDP and health expenditure (HE).

A quality assessment was conducted on all the included studies, utilizing the checklist formulated by Larg and Moss [16]. This checklist comprises three domains: analytic framework, methodology and data, and analysis and reporting. The quality assessment was independently corroborated by two reviewers. In case of any discrepancies in the quality assessment, resolution was ensured through consensus or consultation with a third reviewer.

### Statistical analysis

To analyze the data, we utilized the cost-of-illness (COI) study approach, which involved categorizing the studies into two groups: top-down studies and bottom-up studies. Top-down studies were defined as population-based methods that estimated costs for a specific country or group of countries, while bottom-up studies were defined as person methods that estimated costs per person [16].

In our methodological approach to the top-down studies, we initially categorized the costs into direct and indirect types. The direct costs comprised both medical and nonmedical expenses, while the indirect costs were related to potential productivity losses stemming from mortality and morbidity. Subsequently, we undertook the adjustment of all costs to the 2020 US dollar value. This was achieved based on the principle of purchasing power parity (PPP), and we utilized the currency conversion factor as recommended by the World Bank for this purpose. We employed the method proposed by Konnopka and König to present the COVID-19 cost to top-down studies. This method, which expresses the costs as a proportion of the gross domestic product (GDP) and health expenditure (HE), eliminates the need for adjustments for inflation or differences in purchasing power [17]. Moreover, we computed the costs using both an unweighted mean and a population-weighted mean.

In the bottom-up studies, a random-effects model was employed for the meta-analysis, with the SMD serving as the measure of effect size. To mitigate the influence of heterogeneity, all costs were converted to 2020 US dollars based on PPP, utilizing the currency conversion factor suggested by the World Bank. The focus of our analysis was a comparison of the direct medical costs of patients admitted to the general ward versus those in ICU. The SMD was calculated as the measure of effect size, with the sample size acting as the weighting factor. Heterogeneity was assessed through Cochran’s Q test and the *I* ^2^ statistic. The *Q*-test, a classical measure with a chi-square distribution, is calculated as the weighted sum of squared differences between individual study effects and the pooled effects across studies. The *I* ^2^ statistic represents the percentage of variation across studies, with threshold values of 25%, 50%, and 75% indicating low, moderate, and high levels of heterogeneity, respectively. To assess possible publication or disclosure bias, we used funnel plots, the Begg-adjusted rank correlation test, and Egger’s test. All statistical analyses were performed using STATA version 14 (Stata Corp, College Station, TX, USA), and *P*-values less than 0.05 were considered as statistically significant.

## Results

The study selection process is illustrated in Figure 1. The search strategy produced 3271 records (Scopus, 1450; PubMed, 1144; Web of Science, 677), from which 1358 duplicates were eliminated. Out of the remaining 1913 articles, a mere 101 satisfied the inclusion criteria and underwent a full-text review. During this full-text screening, 74 articles were excluded for various reasons, resulting in a final selection of 27 studies included in the systematic review. Among these, 20 were bottom-up studies [7, 10, 18–35], and 7 were top-down studies [36–42].

### Characteristics of included studies

Table 1 presents the general characteristics of the included studies. Out of the 27 studies, 5 were conducted in the USA; 5 in China; 2 each in Spain, Brazil, South Korea, and India; and 1 each in Italy, South Africa, the Philippines, Greece, Iran, Kenya, Nigeria, and the Kingdom of Saudi Arabia. Based on the methodology employed, 20 studies were categorized as bottom-up studies and seven as top-down studies.

**Table 1:** General characteristics of studies meeting inclusion criteria

Study	Country (years of costing)	Type of assessed cost		
		Direct medical cost	Direct nonmedical cost	Indirect cost
Top-down studies				
Viscusi et al. (2021) [36]	USA (2020)	No	No	Yes
Santos et al. (2021) [37]	Brazil (2020)	Yes	No	No
Zhao et al. (2021) [38]	China (2019)	Yes	Yes	Yes
John et al. (2021) [39]	India (2020)	No	No	Yes
Nurchis et al. (2020) [40]	Italy (2020)	No	No	Yes
Gonzalez Lopez et al. (2020) [41]	Spain (2020)	Yes	Yes	Yes
Debone et al. (2020) [42]	Brazil (2020)	No	No	Yes
Bottom-up studies				
Jeck et al. (2021) [18]	Germany (2020)	Yes	No	No
Kotwani et al. (2021) [19]	India (2021)	Yes	Yes	Yes
Tsai et al. (2021) [20]	USA (2020)	Yes	No	No
Nguyen et al. (2021) [35]	USA (2021)	Yes	No	No
Weiner et al. (2021) [21]	USA (2020)	Yes	No	No
Fusco et al. (2021) [22]	USA (2020)	Yes	No	No
Edoka et al. (2021) [23]	South Africa (2020)	Yes	No	No
Tabuñar et al. (2021) [24]	Philippines (2020)	Yes	No	No
Zhao et al. (2021) [25]	China (2020)	Yes	No	No
Maltezou et al. (2021) [10]	Greece (2020)	Yes	Yes	Yes
Ghaffari Darab et al. (2021) [43]	Iran (2020)	Yes	No	Yes
Barasa et al. (2021) [27]	Kenya (2020)	Yes	No	No
Seon et al. (2021) [28]	South Korea (2020)	Yes	No	No
Banke-Thomas et al. (2021) [29]	Nigeria (2020)	Yes	Yes	Yes
Li et al. (2020) [30]	China (2020)	Yes	No	No
Jin et al. (2020) [7]	China (2019)	Yes	No	Yes
Kirigia et al. (2020) [31]	China (2020)	No	No	Yes
Lee et al. (2020) [32]	South Korea (2020)	Yes	No	No
Khan et al. (2020) [33]	Kingdom of Saudi Arabia (2020)	Yes	No	No
Romero et al. (2020) [34]	Spain (2020)	Yes	Yes	Yes

Among the seven top-down studies, only three calculated direct medical costs [37, 38, 41], two studies examined the direct nonmedical costs [38, 41], and all but Santos et al. [37], who did not report these costs, calculated indirect costs. Of the 20 bottom-up studies, all but 1 study [31] assessed the direct medical costs. Only four studies calculated the direct nonmedical costs [10, 19, 29, 34], and seven studies reported the indirect costs [7, 10, 19, 26, 29, 31, 34].

Table 2 presents the specific characteristics of the top-down studies. These studies indicate that the direct costs of COVID-19 span from US $860 million to US $8,657 million, while indirect costs range from US $610 million to US $5,500,000 million. On average, top-down studies estimate the direct costs associated with COVID-19 to constitute 2.73% and 0.39% of healthcare expenditures, based on unweighted and weighted means, respectively. The results also reveal that, on average, indirect costs account for 10.53% of GDP, with a range of 0.02 to 30.90%. Furthermore, the total cost estimated by top-down studies comprises 85.91% of healthcare expenditure and 9.13% of GDP.

**Table 2:** Specific characteristics of the top-down studies and results of composite analysis

Study	Country	Direct cost ($US PPP^a^)	Indirect cost ($US PPP^a^)	Direct cost (% HE)	Direct cost (% GDP)	Indirect cost (% GDP)	Total cost (% HE)^b^	Total cost (% GDP)
Viscusi et al. (2021) [36]	USA	-	5,500,000	-	-	26.32	157^a^	26.32
Santos et al. (2021) [37]	Brazil	951.4	-	0.69^a^	0.07	-	0.69	0.07
Zhao et al. (2021) [38]	China	860.1	629,559.1	0.11^a^	0.006	4.28	80.03	4.28
John et al. (2021) [39]	India	-	658.6	-	-	0.02	0.82	0.02
Nurchis et al. (2020) [40]	Italy	-	610.7	-	-	0.03	0.37	0.03
Gonzalez Lopez et al. (2020) [41]	Spain	8657.7	396,020	7.40^a^	0.68	30.90	345.80	31.58
Debone et al. (2020) [42]	Brazil	-	23,065.1	-	-	1.60	16.65	1.60
Unweighted mean				2.73	0.25	10.53	85.91	9.13
Weighted (by population) mean				0.39	0.03	4.81	50.89	4.54

^a^All cost converted to $US 2020 (PPP), the number is ($ million), ^b^health expenditure is for 2019

Table 3 outlines the specific characteristics of the bottom-up studies. Excluding two studies [23, 27], all reported their sample sizes, which varied from 9 to 1,470,721. The mean estimate of direct medical costs ranged from US $1264 to US $79,315. Two studies reported values for direct nonmedical costs [19, 29], with means of US $25 and US $71. The mean estimate of indirect costs ranged from US $187 to US $689,556.

**Table 3:** Specific characteristics of bottom-up studies and results of composite analysis

Study	Country	Sample size	Direct medical cost (mean, $US PPP*)	Direct nonmedical cost (mean, $US PPP*)	Indirect cost (mean, $US PPP*)
Jeck et al. (2021) [18]	Germany	105	25,244	-	-
Kotwani et al. (2021) [19]	India	138	1264	25	187
Tsai et al. (2021) [20]	USA	268,706	21,752	-	-
Nguyen et al. (2021) [35]	USA	17,456	54,156	-	-
Weiner et al. (2021) [21]	USA	1,470,721	1701	-	-
Fusco et al. (2021) [22]	USA	173,942	24,571	-	-
Edoka et al. (2021) [23]	South Africa	NA	1908	-	-
Tabuñar et al. (2021) [24]	Philippines	691	12,236	-	-
Zhao et al. (2021) [25]	China	100	79,315	-	-
Maltezou et al. (2021) [10]	Greece	254	1571	-	4025
Ghaffari Darab et al. (2021) [43]	Iran	477	3755	-	4145
Barasa et al. (2021) [27]	Kenya	NA	4293	-	-
Seon et al. (2021) [28]	South Korea	7969	7814	-	-
Banke-Thomas et al. (2021) [29]	Nigeria	9	2551	71	1144
Li et al. (2020) [30]	China	70	11,256		
Jin et al. (2020) [7]	China	81,879/915,824	5264	-	689,556
Kirigia et al. (2020) [31]	China	2595	-	-	587,290
Lee et al. (2020) [32]	South Korea	145	3002	-	-
Khan et al. (2020) [33]	Kingdom of Saudi Arabia	1422	30,021	-	-
Romero et al. (2020) [34]	Spain	198	2258	-	-

*All costs converted to $US 2020 (PPP)

### Meta-analysis results

**Fig. 2:**
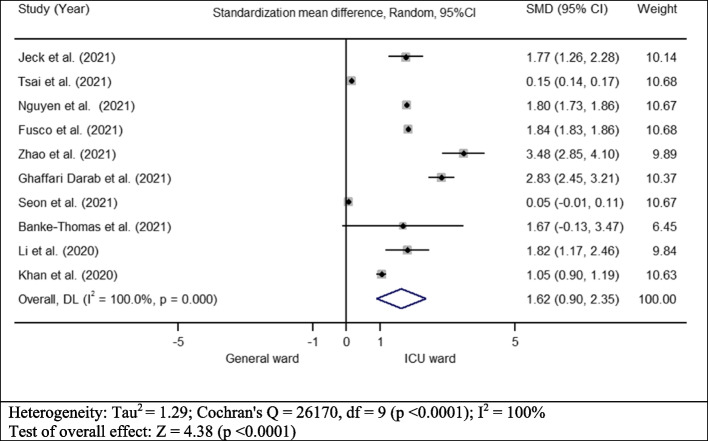
Mean direct medical cost for patient with COVID-19 based on disease severity

The results of the meta-analysis for the direct medical costs are shown in Figure 2. The results indicate a significant association between the mean cost of direct medical services and the inpatient ward. Specifically, the analysis yielded a standardized mean difference (SMD) of 1.62 (*CI*: 0.9–2.35) with a substantial degree of heterogeneity (*Q* = 26170, *p* < 0.0001; *I* ^2^ = 100%).

### Assessment of publication bias

**Fig. 3:**
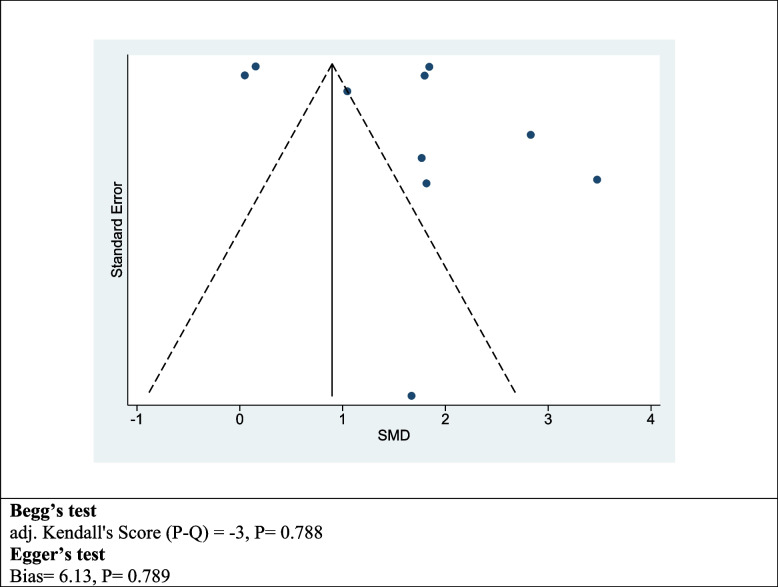
The funnel plots, Begg’s test, and Egger’s test to assessment of publication bias for included studies that assessed the direct medical costs of patients hospitalized in the general ward versus those in the intensive care unit (ICU)

Figure 3 presents the information related to publication bias. The funnel plot, constructed from the studies included, does not suggest the presence of potential publication bias. Moreover, the application of Begg’s and Egger’s tests in the statistical analysis resulted in P-values of 0.788 and 0.789, respectively, indicating an absence of significant bias.

## Discussion

This investigation represents the initial systematic review and meta-analysis conducted on the topic of the global economic impact of COVID-19. Furthermore, it is the first study to evaluate economic burden research related to COVID-19 using both top-down and bottom-up approaches, and it has conducted a meta-analysis of medical direct expenses based on hospitalization wards. In general, studies examining the economic impact of COVID-19 are scarce, with a greater proportion of studies employing a bottom-up approach. More than 30% of these studies were conducted in the USA and China. Patients admitted to the ICU ward exhibited higher costs than those admitted to the general ward.

Admission to the ICU significantly escalated the medical expenditure associated with COVID-19 treatment. This study discovered that the medical costs for COVID-19 patients in the ICU were approximately twice as high as those for patients in general wards, with a range from 0.05 to 3.48 times higher. This finding aligns with existing literature, which suggests that ICU patients with COVID-19 are more likely to require expensive treatments such as mechanical ventilation and extracorporeal membrane oxygenation, compared to those in general wards [44, 45]. Consistent with this, other studies have reported an increase in medical expenditures with the hospitalization of COVID-19 patients in the ICU. For instance, a study conducted in the USA found a fivefold increase in costs for patients in the ICU who required invasive mechanical ventilation (IMV), compared to those not in the ICU or without IMV [22]. Similarly, a study in China reported a 2.5-fold increase in costs for severe COVID-19 patients compared to mild cases [30]. Given the elevated medical costs associated with treating COVID-19 patients in the ICU or those with severe symptoms, health policymakers must concentrate on implementing programs that promote early diagnosis. Consequently, healthcare providers could initiate treatment at an earlier stage, potentially reducing the severity of the disease and associated costs.

Our research indicates that significant variations in estimated costs would be observed if these costs were reported in PPP, particularly in relation to direct medical expenses. The lowest value was calculated in India, amounting to US $1264, while the highest value was observed in the USA, reaching US $54,165. Furthermore, the calculated medical costs varied across countries. For example, in the USA, direct medical expenditures ranged from US $1701 to US $54,156 [21, 35]. In contrast, in China, the reported costs fluctuated between US $5264 and US $79,315 [7, 25]. Several factors contribute to this variation in the estimation of direct medical costs. Primarily, direct medical costs cover a spectrum of services, including diagnosis, medication, consumables, inpatient care, and consultation services. Consequently, each study may have estimated the direct medical costs for a subset or the entirety of these services, leading to differences in the estimated costs. For instance, Nguyen et al. demonstrated a nearly threefold increase in direct costs for COVID-19 patients managed with extracorporeal membrane oxygenation (ECMO) compared to patients not receiving ECMO [35]. This highlights the impact of specific treatments on the overall cost. Secondly, the sample size may vary between studies, resulting in different cost estimates. Larger sample sizes typically provide more accurate and reliable estimates, but they also require more resources to collect and analyze. Lastly, the studies may have estimated costs for patients with varying conditions, such as those in acute status, patients hospitalized in general wards, or those admitted to ICU wards.

In addition to direct medical expenditures, the indirect costs arising from productivity losses due to COVID-19 have substantial societal implications. This study discovered that direct medical expenses attributable to COVID-19 varied from US $860 million (representing 0.11% of China’s healthcare expenditure) as reported by Zhao et al. [38] in China to US $8657 million (equivalent to 7.4% of Spanish healthcare expenditure) as reported by Gonzalez Lopez et al. [41] in Spain. On a global scale, direct medical costs due to COVID-19 constituted 2.73% of healthcare expenditure and 0.25% of GDP. The results also unveiled that the indirect costs of the COVID-19 pandemic impacted different countries to varying extents. The minimum value of indirect costs was estimated in Italy [40] and India [39] at US $610 million and US $658 million, respectively. Interestingly, when reported as a percentage of GDP, India had a lower cost (0.02% of GDP) compared to China (0.03% of GDP). The maximum value of indirect costs was calculated in the USA at US $5,500,000 million, which accounted for approximately 26.32% of the USA’s GDP [36]. Despite the numerical value of indirect costs being lower in Spain than in the USA and China, it represented a higher percentage of GDP (30.90%). The resulting pooled estimate indicated that the indirect costs due to COVID-19 were responsible for 10.53% of global GDP. The review underscores the significant economic repercussions of COVID-19. The total costs in the USA accounted for about 157% of healthcare expenditure and 26% of GDP, in China for 80% of healthcare expenditure and 4.28% of GDP, and in Spain for approximately 345% of healthcare expenditure and 32% of GDP. Globally, the total costs of COVID-19 accounted for about 86% of healthcare expenditure and 9.13% of GDP. This highlights the profound economic impact of the pandemic on both healthcare systems and economies worldwide.

### Strengths and limitation

Our study possesses several significant strengths. It is the inaugural meta-analysis of the worldwide costs associated with COVID-19, supplementing a systematic review conducted by Richards et al. on the economic burden studies of COVID-19 [12]. A considerable number of studies was conducted in the USA and China, but our analysis also incorporated studies from other high- and low-income countries, potentially enhancing the generalizability of our findings. Recognizing that economic burden studies often display significant heterogeneity, we endeavored to minimize this by distinguishing between bottom-up and top-down studies and standardizing currencies to US dollars in terms of PPP.

However, our study is not without limitations. As is typical with all meta-analyses of economic burden studies, the most substantial limitation is heterogeneity. This heterogeneity can originate from various factors, including differences in study design, the range of services included in individual studies, the year of estimation, the currencies used for estimation, the study population, among other factors. Our systematic review only incorporated studies that estimated costs for an actual population, thereby excluding a wide array of studies on the economic burden of COVID-19 that employed modeling techniques. Future research could potentially conduct systematic reviews and meta-analyses on cost estimation modeling studies for COVID-19. Lastly, while no publication bias was detected through statistical analysis, our study was limited to papers written in English. As a result, numerous papers published in other languages were inevitably excluded.

## Conclusion

Our research indicates that the COVID-19 pandemic has imposed a substantial economic strain worldwide, with the degree of impact varying across nations. The quantity of studies examining the economic repercussions of COVID-19 is limited, with a majority employing a bottom-up methodology. The indirect costs ascribed to COVID-19 constituted 10.53% of the global GDP. In total, the costs linked to COVID-19 represented 9.13% of GDP and 86% of healthcare spending. Moreover, our meta-analysis disclosed that the direct medical expenses for COVID-19 patients in the ICU were almost twice those of patients in general wards. The results of our research, along with those of others, underscore the pivotal role of economic outcomes in the post-COVID-19 era for societies and families. Consequently, it is imperative for policymakers and health administrators to prioritize and pay greater attention to economic programs.

### Supplementary information

**Additional file 1.** Search strategies

## Data Availability

Data sharing is not applicable as no new data were generated during the study. The data analysis file during this study is available from the corresponding author on reasonable request.
