# Supplementary material for: The global economic burden of COVID-19 disease: a comprehensive systematic review and meta-analysis
Source: Syst Rev. 2024 Feb 16;13:68. doi: 10.1186/s13643-024-02476-6 (PMC10870589; doi:10.1186/s13643-024-02476-6)
Supplement: Supplementary file 1 — Additional file 1. Search strategies [file 13643_2024_2476_MOESM1_ESM.docx]

| Database name | Search Startegy |
| --- | --- |
| PubMed | (("COVID-19"[MeSH Terms] OR "SARS-CoV-2"[MeSH Terms] OR "Coronaviridae"[MeSH Terms] OR "COVID-19"[Title/Abstract] OR "COVID19"[Title/Abstract] OR "COVID-2019"[Title/Abstract] OR "COVID2019"[Title/Abstract] OR "SARS-CoV-2"[Title/Abstract] OR "SARS CoV 2"[Title/Abstract] OR "coronavirus*"[Title/Abstract] OR Coronaviridae[Title/Abstract] OR Coronavirinae [Title/Abstract] OR "2019-nCoV"[Title/Abstract] OR "2019 nCoV"[Title/Abstract] OR "HCoV-19"[Title/Abstract] OR "HCoV 19"[Title/Abstract] OR "nCoV-2019"[Title/Abstract] OR "nCoV 2019"[Title/Abstract] OR "corona virus"[Title/Abstract] OR "sarscov*"[Title/Abstract] OR "ncov*"[Title/Abstract] OR "new cov"[Title/Abstract] OR "novel cov"[Title/Abstract])) AND ((Cost of Illness [MeSH Terms] OR Health Expenditures [MeSH Terms] OR "Costs and Cost Analysis"[ MeSH Terms] OR "Medical cost"[Title/Abstract] OR "nonmedical cost"[Title/Abstract] OR "non-medical cost"[Title/Abstract] OR "direct cost"[Title/Abstract] OR "indirect cost"[Title/Abstract] OR "productivity cost"[Title/Abstract] OR "morbidity cost"[Title/Abstract] OR "mortality cost"[Title/Abstract] OR "economic cost"[Title/Abstract] OR "financial cost"[Title/Abstract] OR "non-economic cost"[Title/Abstract] OR "noneconomic cost"[Title/Abstract] OR "cost analysis"[Title/Abstract] OR "cost of illness"[Title/Abstract] OR "health expenditure"[Title/Abstract] OR "economic burden"[Title/Abstract] OR expenditure*[Title/Abstract] OR "productivity lost"[Title/Abstract])) |
| Web of Science | TS=(covid-19 OR sars-cov-2 OR coronaviridae OR covid19 OR covid-2019 OR covid2019 OR "SARS CoV 2" OR coronavirus* OR coronavirinae OR 2019-ncov OR "2019 nCoV" OR hcov-19 OR "hcov 19" OR ncov-2019 OR "nCoV 2019" OR "corona virus" OR sarscov* OR ncov* OR "new cov" OR "novel cov") AND TS = ("Medical cost" OR "nonmedical cost" OR "non-medical cost" OR "direct cost" OR "indirect cost" OR "productivity cost" OR "morbidity cost" OR "mortality cost" OR "economic cost" OR "financial cost" OR "non-economic cost" OR "noneconomic cost" OR "cost analysis" OR "cost of illness" OR "health expenditure*" OR "economic burden" OR expenditure* OR "productivity lost")  Indexes=SCI-EXPANDED, SSCI, A&HCI, CPCI-S, CPCI-SSH, BKCI-S, BKCI-SSH, ESCI Timespan=All years |
| Scopus | ( TITLE-ABS-KEY ( covid-19 OR sars-cov-2 OR coronaviridae OR covid19 OR covid-2019 OR covid2019 OR "SARS CoV 2" OR coronavirus* OR coronavirinae OR 2019-ncov OR "2019 nCoV" OR hcov-19 OR "hcov 19" OR ncov-2019 OR "nCoV 2019" OR "corona virus" OR sarscov* OR ncov* OR "new cov" OR "novel cov" ) ) AND ( TITLE-ABS-KEY ( "Medical cost" OR "nonmedical cost" OR "non-medical cost" OR "direct cost" OR "indirect cost" OR "productivity cost" OR "morbidity cost" OR "mortality cost" OR "economic cost" OR "financial cost" OR "non-economic cost" OR "noneconomic cost" OR "cost analysis" OR "cost of illness" OR "health expenditure*" OR "economic burden" OR expenditure* OR "productivity lost" ) ) |
